# Transient Glycocalyx Remodeling by Intravenous Hyaluronidase in Atherosclerosis: A Hypothesis-Generating Review

**DOI:** 10.3390/pathophysiology33020026

**Published:** 2026-04-10

**Authors:** Andreas Pfützner, Tobias Gantner, Harald Burgard, Tilman Steinmeier, Eduard Stappler, Julia Jantz, Petra Wiechel

**Affiliations:** 1 Pfützner Science & Health Institute, PSHI Praxis GmbH, Haifa-Alle 18, D-55128 Mainz, Germany; julia.jantz@pfuetzner-mainz.com; 2 Department of Biotechnology & Bioinformatics, Technische Hochschule Bingen, D-55411 Bingen am Rhein, Germany; 3 Institute for Digital Technology in Medicine and Dentistry, Institut Supérieur de Formation Continue, Schuttrange, L-5359 Luxembourg, Luxembourg; 4 Healthcare Futurists GmbH, D-50935 Cologne, Germany; tobias.gantner@healthcarefuturists.com; 5 Practice for Integrative Environmental Medicine, D-66787 Wadgassen, Germany; burgard@med-select.de; 6 Biologicum—Zentrum für Umweltmedizin, D-20251 Hamburg, Germany; til.steinmeier@icloud.com; 7 MedDentcon GmbH, D-50667 Cologne, Germany; dr.stappler@medentcon.de; 8 Swiss Mountain Clinic, CH-6540 Castaneda, Switzerland; petra.wiechel@swissmountainclinic.com

**Keywords:** atherosclerosis, hyaluronidase, glycocalix remodeling, reverse cholesterol transport, vascular barrier, endothelial dysfunction

## Abstract

Atherosclerosis remains the leading cause of death worldwide and imposes a major healthcare burden. Physiologically, elimination of cholesterol from the arterial wall depends on reverse cholesterol transport (RCT). RCT requires access to HDL and apolipoprotein A-I (ApoA-I) to lesional macrophages/foam cells. The endothelial glycocalyx is a dynamic and injury-sensitive layer of proteoglycans and glycosaminoglycans (including hyaluronan). It contributes to vascular barrier properties, leukocyte adhesion, mechanotransduction, and macromolecular transport. In atherosclerosis, glycocalyx structure and function are altered; this may facilitate entry/retention of atherogenic lipoproteins and may also alter transport conditions relevant to cholesterol efflux pathways. This article presents a mechanistic hypothesis: short, transient, systemic hyaluronidase exposure could temporarily remodel glycocalyx/extracellular matrix components and thereby facilitate conditions permissive for regulated transport processes relevant to RCT. However, the proposed link between glycocalyx remodeling and improved lesional cholesterol efflux remains theoretical. Direct in vivo evidence that the endothelial glycocalyx is a dominant barrier limiting HDL- or ApoA-I-mediated cholesterol efflux from plaque macrophages is currently limited. Moreover, glycocalyx degradation is widely associated with endothelial dysfunction, increased permeability, inflammation, and thrombosis, all of which could aggravate rather than ameliorate atherosclerosis. Human pharmacokinetic data indicate a very short plasma half-life of circulating hyaluronidase activity, suggesting that any systemic enzymatic effect is brief. Nevertheless, the biological consequences of repeated degradation–regeneration cycles, especially in high-risk states such as diabetes, inflammation, oxidative stress, or chronic kidney disease, remain incompletely understood. Evidence supporting clinical benefit in atherosclerosis is currently limited to heterogeneous animal experiments, historical uncontrolled reports, and a small number of anecdotal case observations, whereas randomized trials have only been performed in other settings such as acute myocardial infarction and do not establish efficacy for plaque regression. We therefore provide a balanced evaluation of knowns, uncertainties, alternative interpretations, potential risks, dosing unknowns, and a translational research agenda including mechanistic preclinical studies, biomarker development, imaging, and carefully designed early-phase clinical investigation.

## 1. Introduction

Atherosclerosis and its clinical sequelae remain the leading causes of morbidity and mortality in Western societies and impose a substantial economic burden on healthcare systems worldwide. Global analyses have shown persistently high incidence and mortality of atherosclerotic cardiovascular disease (ASCVD) in recent decades, with substantial regional variation and high burden in Europe [1,2]. The economic consequences are similarly profound; in large matched-cohort analyses, individuals with ASCVD incur several-fold higher healthcare costs and major indirect costs due to work absence [3].

Current therapeutic strategies reduce risk primarily by addressing modifiable factors (lipids, blood pressure, diabetes control, smoking) [4,5,6] and, in advanced disease, employ revascularization procedures [4,5]. Several risk determinants are partly non-modifiable. Among these, genetically determined elevated lipoprotein(a) is a recognized causal risk factor [7]. Intensive lipid-lowering treatment, including contemporary combination regimens and PCSK9 inhibitor-based approaches, has shown evidence of plaque regression in selected human studies [Lit]. Nevertheless, routine clinical care does not currently include an intervention specifically designed to directly facilitate cholesterol mobilization from established arterial lesions through transient remodeling of the vascular interface. Notably, current guidelines focus on prevention and risk-factor control, while interventions explicitly aiming to remove cholesterol deposits from arterial walls are not established in routine clinical care [8,9,10].

At the mechanistic level, atherosclerosis can be viewed as a failure of coordinated endogenous repair processes, including lipid clearance, inflammation resolution, endothelial restoration, and cellular emigration [11]. Regression of lesions is biologically plausible and has been demonstrated in experimental settings [12,13]. However, translation into reliable clinical plaque removal or plaque normalization remains an unmet need.

This review does not claim established clinical efficacy of intravenous hyaluronidase treatment for atherosclerosis. Rather, it develops a hypothesis linking (i) endothelial glycocalyx dysfunction, (ii) altered transport conditions relevant to HDL/ApoA-I access and macrophage cholesterol efflux, and (iii) possible transient remodeling by hyaluronidase, while explicitly addressing uncertainties, alternative interpretations, risks, dosing unknowns, and the level of evidence supporting each component.

## 2. Endogenous Cholesterol Clearance from the Arterial Wall: Reverse Cholesterol Transport

**Figure 1:**
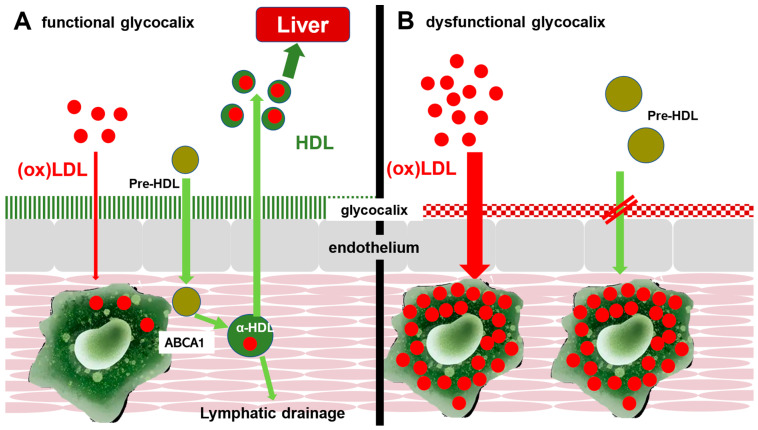
Role of the endothelial glycocalyx in lipoprotein transport and reverse cholesterol transport (conceptual model). (**A**) Under physiological conditions, the endothelial glycocalyx regulates macromolecular flux and supports controlled interactions at the vascular interface; HDL/ApoA-I access to subendothelial macrophages can facilitate cholesterol efflux via ABCA1/ABCG1 and contribute to RCT. LDL entry into the arterial wall is constrained. (**B**) In vascular inflammation, glycocalyx structure/function is altered. This may facilitate increased LDL penetration/retention and may reduce effective access of HDL/ApoA-I to lesional foam cells, contributing to an imbalance between influx and efflux that promotes cholesterol accumulation and lesion progression. Oxidative modification of LDL occurs within the intimal environment after entry and retention. The figure illustrates a hypothesis-driven conceptual framework rather than an established pathway.

Removal of cholesterol from the arterial wall is mediated by reverse cholesterol transport (RCT) [14,15]. In this pathway, macrophages/foam cells efflux excess cholesterol via ATP-binding cassette transporters ABCA1 and ABCG1 to ApoA-I and nascent HDL particles [16,17]. HDL then transports cholesterol to the liver for biliary secretion or conversion into bile acids [15,18]. RCT therefore represents a central physiological route for clearing cholesterol from lesions and eliminating it from the body (Figure 1A). Reverse cholesterol transport from the arterial wall is likely not confined only to direct return of cholesterol into the plasma compartment. Adventitial and lymphatic pathways have also been implicated in sterol and immune cell trafficking from atherosclerotic lesions. Therefore, impaired lymphatic drainage may represent an additional mechanism of defective vascular cholesterol clearance, potentially acting in parallel with endothelial transport abnormalities [19,20,21]. At the same time, atherosclerosis is not solely a disease of impaired cholesterol removal. Contemporary understanding emphasizes the interaction of lipid retention, chronic vascular inflammation, endothelial dysfunction, smooth muscle cell phenotypic modulation, extracellular matrix remodeling, thrombosis-related pathways, and lesion-specific immune-cell dynamics. The present hypothesis focuses on one transport-related aspect of this broader biology and should be interpreted within that larger context.

## 3. Conceptual Model of Glycocalyx Dysfunction

The endothelial glycocalyx is a gel-like, carbohydrate-rich layer composed of proteoglycans, glycosaminoglycans (including heparan sulfate and hyaluronan), adsorbed plasma proteins, and associated molecules [22,23]. It contributes to barrier regulation, shear sensing, anti-inflammatory and antithrombotic signaling, and modulation of leukocyte adhesion [24,25]. In principle, glycocalyx structure can influence macromolecular and lipoprotein movement between blood and the intimal compartment.

During vascular inflammation, glycocalyx composition and thickness are altered through enzymatic activity (e.g., heparanase, metalloproteinases, hyaluronidases), reactive oxygen species, and cytokines [24,25]. These changes are associated with increased permeability and altered leukocyte interactions.

We hypothesize that, in atherosclerosis, glycocalyx dysfunction may create a maladaptive vascular interface that allows enhanced influx and retention of atherogenic particles and may also alter effective access of HDL and ApoA-I to lesional foam cells, thereby reducing functional RCT within the arterial wall. However, this remains a conceptual model. Direct in vivo evidence that the endothelial glycocalyx represents a dominant barrier limiting HDL-mediated cholesterol efflux from arterial macrophages is currently limited. Conversely, glycocalyx degradation is well recognized to promote endothelial dysfunction, leukocyte adhesion, permeability, and inflammatory signaling. These changes could theoretically exacerbate plaque biology rather than improve it.

Accordingly, the proposed mechanism should be regarded as one testable interpretation among several competing possibilities, not as an established explanation of impaired RCT in human atherosclerosis. Validation would require dedicated experimental systems, including imaging methods for glycocalyx integrity, tracer-based assessment of HDL/ApoA-I penetration into lesional tissue, lesion-level cholesterol efflux assays, and quantitative biomarkers of hyaluronan shedding and fragment generation.

The loss of the barrier function leads to enhanced LDL penetration into the intima and promotion of plaque formation. In parallel, HDL/ApoA-I access to foam cells is impaired, disrupting the “return pathway” for cholesterol. Cholesterol efflux from macrophages and other lesional cells is mediated by a coordinated network that includes ABCA1, ABCG1, and SR-BI. Whereas ABCA1 promotes cholesterol transfer to lipid-poor ApoA-I and ABCG1 facilitates efflux to more mature HDL particles, SR-BI also contributes to cholesterol exchange and to the overall efficiency of reverse cholesterol transport [26,27]. Accordingly, any impairment of vascular cholesterol clearance is likely to reflect dysfunction at multiple transport steps rather than a single pathway. If these receptor-mediated interactions (e.g., ABCA1, SR-BI) are compromised, the efflux capacity is further impaired. In addition, proinflammatory glycocalyx fragments, such as hyaluronan and heparan sulfate breakdown products, act as danger-associated molecular patterns that attract monocytes, thereby increasing macrophage recruitment and net cholesterol accumulation [28,29]. This leads to the morphological consequence that the glycocalyx shifts from a permeable, regulated interface into a proinflammatory barrier: facilitating LDL entry while blocking cholesterol removal by HDL. The equilibrium between influx and efflux is disrupted (influx > efflux), driving cholesterol retention and the progression of atherosclerotic lesions (Figure 1B.).

## 4. Hyaluronan Biology and Metabolism: What Must Be Considered

Because the proposed mechanism involves hyaluronidase and glycocalyx hyaluronan, any therapeutic hypothesis must account for hyaluronan metabolism, turnover, clearance, and fragment biology. Hyaluronan (HA) is synthesized at the plasma membrane by hyaluronan synthases (HAS1-3) and is abundant in extracellular matrices and pericellular coats. HA is present in vascular tissues and contributes to hydration, matrix organization, and cell signaling [30]. HA turnover is dynamic, and turnover rates can differ substantially across tissues and disease states [31].

HA can be degraded enzymatically (e.g., hyaluronidases) and non-enzymatically (oxidative cleavage). Importantly, HA fragments can have biological activities distinct from high-molecular-weight HA and may function as danger-associated molecular patterns that influence immune cell recruitment, toll-receptor signaling, and endothelial activation [30,32]. Circulating HA and fragments are cleared via receptor-mediated pathways in the liver, spleen, and lymphatic systems. These processes may be altered in inflammation, metabolic disease, and organ dysfunction [33,34].

Any systemic hyaluronidase administration could, in principle, transiently alter HA integrity in glycocalyx and extracellular matrix compartments. It could generate HA fragments with potential signaling. It could influence vascular permeability, leukocyte adhesion, and inflammatory signaling. It could also interact with disease states that already feature glycocalyx impairment. For these reasons, fragment biology and clearance must be considered central questions of both mechanism and safety rather than ancillary issues.

## 5. Glycocalyx Degradation and Regeneration After i.v. Hyaluronidase Application

Experimental models show that enzymatic degradation (including hyaluronidase exposure) can rapidly thin/collapse glycocalyx structures, with associated increases in permeability and leukocyte adhesion [22,24]. Human pharmacokinetic studies indicate that circulating enzymatic activity following intravenous hyaluronidase is short-lived, with a reported half-life on the order of minutes (~2–10 min) due to rapid neutralization by plasma inhibitors [35,36]. This supports the plausibility that systemic effects may be brief.

Regeneration of glycocalyx components has been observed after injury; recovery can begin within hours and may approach baseline within one to three days in experimental settings [37,38,39]. However, regeneration kinetics are heterogeneous and can be delayed by inflammatory/metabolic stressors [22] (Table 1). Recovery data from experimental systems cannot be assumed to generalize to repeated systemic enzymatic exposure in patients with advanced vascular disease.

Intravenous hyaluronidase administration can enzymatically degrade the endothelial glycocalyx within minutes, but because the enzyme’s plasma half-life is only a few minutes, any systemic effect is transient. The glycocalyx begins to regenerate within hours and is usually restored within 1–3 days under healthy conditions [37,38,39].

## 6. Impact of Repeated Short-Term i.v. Hyaluronidase Infusion on the Arterial Wall

At present, it remains unknown whether repeated, transient systemic hyaluronidase exposure leads to cumulative changes in glycocalyx composition, endothelial phenotype, or vascular bed–specific injury in humans with ASCVD. In addition, HA-fragment generation could potentially amplify inflammation or thrombosis risk in susceptible settings, such as diabetes, active infection, or advanced plaque instability. Repeated remodeling may therefore have heterogeneous effects—beneficial in some settings, neutral in others and harmful in still others—depending on local pathology and regeneration capacity.

If transient enzymatic remodeling were ever to facilitate RCT, this would most plausibly occur only under narrowly defined conditions in which barrier and transport properties are altered briefly and followed by reconstitution toward a more functional vascular interface. Even this scenario remains unproven. An equally plausible interpretation is that repeated glycocalyx disruption could worsen vascular inflammation by increasing endothelial permeability, leukocyte adhesion, monocyte recruitment, and local matrix signaling within the plaque environment.

## 7. Preclinical Evidence for i.v. Hyaluronidase Infusion

Ozegowski et al. reported anti-atherosclerotic effects of microbial hyaluronate lyase in an animal model using repeated intravenous administrations, with treated animals described as lacking anatomical/histological signs of atherosclerosis compared with placebo [40]. While provocative, translation is limited not only by the animal model itself but also by the substantial biological differences between microbial hyaluronate lyases and mammalian hyaluronidases. These enzyme classes differ in catalytic properties, substrate cleavage characteristics, fragment-generation patterns, and potentially in downstream biological effects. Accordingly, these data support biological plausibility only in a narrow sense and cannot be interpreted as evidence of clinical efficacy.

Preclinical work also provides signals of potential adverse consequences. In apolipoprotein E-deficient mice, Meuwese et al. showed that chronic hyaluronidase infusion degraded the endothelial surface layer and induced proteinuria while also altering plaque composition in ways that may be relevant to plaque phenotype [41]. These findings are not merely a caveat; they represent an important biological warning signal. They suggest that sustained or cumulative glycocalyx degradation may cause off-target renal and vascular harm. Exposure duration, cumulative burden, vascular-bed susceptibility, and recovery capacity are therefore likely critical determinants of net effect.

Additional older experimental work explored hyaluronidase in arteriosclerosis and atherosclerosis, including early Soviet-era studies, but these reports generally lack modern study design, mechanistic endpoints, and standardized lesion quantification, limiting interpretability for contemporary clinical translation [42]. The preclinical literature does, therefore, not provide a uniform “benefit signal.” Rather, it supports (i) plausibility that hyaluronan/glycocalyx manipulation can influence vascular biology, while simultaneously (ii) identifying credible risks when exposure is prolonged.

Because the hypothesis remains in an early stage, future work should include basic mechanistic preclinical studies that characterize the cellular and vascular consequences of transient systemic hyaluronidase exposure. Such studies should assess endothelial glycocalyx architecture, endothelial barrier integrity, leukocyte–endothelial interactions, HA-fragment generation, inflammatory signaling, thrombogenic readouts, and lesion-level cholesterol efflux in relevant experimental systems. These data are essential to inform later translational development, including dose selection, infusion intervals, monitoring strategies, and patient selection.

## 8. Clinical Observations

Several human case reports describe temporal associations between serial intravenous hyaluronidase infusions and improvements in perfusion or clinical outcomes in severe vascular disease contexts [43,44,45]. These reports are uncontrolled, subject to confounding by co-interventions, regression to the mean, and natural fluctuation of symptoms, and cannot establish causality or generalizability.

Beyond these modern case reports, there is also historical clinical experience with intra-arterial or intravenous hyaluronidase-containing regimens in peripheral or cerebral vascular disease. For example, Elder et al. reported intra-arterial hyaluronidase use in severe peripheral arterial disease (case report format) [46]. In addition, several older German-language series described intravenous hyaluronidase (often combined with magnesium preparations) in peripheral arterial disease, reporting symptomatic or functional improvements but without modern control groups or structured adverse-event adjudication [47,48,49]. Similarly, intra-arterial infusion approaches were described for intracranial perfusion disorders in small, uncontrolled cohorts [50].

Taken together, these cases suggest that serial intravenous hyaluronidase infusions may induce clinically relevant improvements in micro- and macrovascular complications by modifying extracellular matrix structures, potentially restoring endothelial glycocalyx function, and facilitating reverse cholesterol transport. Collectively, these reports suggest procedural feasibility and short-term tolerability in heterogeneous vascular contexts, but they do not establish efficacy and provide limited high-quality safety granularity by modern standards.

Randomized trials of systemic hyaluronidase have been conducted primarily in acute myocardial infarction. A double-blind trial by Cairns et al. evaluated intravenous hyaluronidase for infarct size limitation and did not show a statistically robust overall clinical benefit, although trends and subgroup signals were discussed in early-treated patients [51].

Additional randomized studies in the early reperfusion era similarly did not demonstrate consistent benefit on major outcomes overall, but some reports suggested more favorable biomarker/infarct-size patterns among patients treated very early after symptom onset [52]. Importantly for the present manuscript’s safety discussion, these AMI trials did not report a major excess of macrovascular adverse events attributable to hyaluronidase under the studied conditions, even at relatively high systemic dosing regimens [53,54]. Importantly, those trials inform feasibility and short-term tolerability only within the setting of acute ischemic injury. They do not validate efficacy for plaque regression or vascular repair in chronic atherosclerosis.

At present, no convincing clinical evidence supports hyaluronidase therapy as an effective treatment for chronic atherosclerosis. The available human data should therefore be interpreted strictly as anecdotal, uncontrolled, and hypothesis-generating.

## 9. Safety Considerations

Although circulating hyaluronidase activity after intravenous administration appears short-lived, with reported plasma half-lives in the range of minutes [30,31], pharmacokinetic brevity should not be equated with biological triviality. Even transient enzymatic exposure can initiate downstream processes that outlast measurable circulating activity, including changes in endothelial barrier properties, leukocyte–endothelial interactions, and generation of bioactive extracellular matrix fragments.

It is important to emphasize that glycocalyx loss is not intrinsically beneficial. On the contrary, it is widely regarded as a hallmark of endothelial injury and vascular dysfunction. Glycocalyx degradation has been associated with increased permeability, inflammatory cell adhesion, disturbed mechanotransduction, and impaired vascular homeostasis [23,55]. Thus, any proposed benefit from transient enzymatic remodeling must be interpreted against the competing possibility that further glycocalyx disruption may aggravate endothelial injury. This duality is central to the present hypothesis and underscores the need for cautious experimental testing.

From a mechanistic perspective, transient degradation of glycocalyx constituents is expected to influence vascular barrier function. Experimental data indicate that glycocalyx thinning can increase permeability and facilitate leukocyte adhesion within minutes [22,24]. Clinically, this translates into a plausible risk of transient vascular leakage and tissue edema during or shortly after exposure. At the same time, the “double barrier” concept emphasizes that the endothelial glycocalyx and the endothelial cell monolayer independently contribute to macromolecular restriction; therefore, glycocalyx disruption alone does not necessarily imply severe barrier failure provided the endothelial monolayer remains intact [31,32]. Susceptibility is likely to vary with comorbidity, disease stage, and concurrent endothelial dysfunction.

Beyond permeability, glycocalyx disruption may promote a pro-inflammatory endothelial phenotype. Enzymatic shedding is associated with increased leukocyte adhesion and endothelial activation in experimental settings [22,24], and hyaluronan shedding is a recognized feature of multiple vascular pathologies, including diabetes, sepsis, and atherosclerosis [30]. A central uncertainty concerns fragment biology: hyaluronan fragments can act as pro-inflammatory signals [29,30], and it remains unclear to what extent systemic hyaluronidase administration in ASCVD patients generates circulating or locally active fragment profiles of sufficient magnitude to be clinically relevant.

The glycocalyx is also implicated in antithrombotic and anticoagulant vascular properties [22,23,30]. Consequently, transient disruption could theoretically shift the local thrombo-inflammatory balance, particularly in high-risk contexts. While such risk remains hypothetical in the absence of systematic data in ASCVD, it argues for cautious patient selection and monitoring in any future study.

Finally, immunogenicity and hypersensitivity risks depend strongly on the enzyme source and formulation. Animal-derived preparations may carry a different allergy and sensitization profile than recombinant products [55,56], and repeated exposure could potentially increase the likelihood of immune reactions. Any future clinical development pathway should therefore incorporate explicit risk mitigation for allergic reactions and immunogenicity, including careful documentation of product type, dosing, and adverse events.

## 10. Dosing Considerations

At present, dosing considerations for systemic hyaluronidase in the context of chronic atherosclerosis must be presented with strict separation between what is supported by existing data and what remains unknown. Human pharmacokinetic studies consistently indicate that circulating enzymatic activity is short-lived, with rapid neutralization in plasma and half-lives reported in the range of minutes [35,36]. Historical randomized experience in acute myocardial infarction provides contextual information on feasibility and acute tolerability of systemic administration across a defined time window and dosing intensity, without a prominent excess safety signal in those trials [51,52,53,54,57].

However, key dosing parameters remain unknown. These include the dose and infusion duration required for any hypothesized vascular effect, the optimal interval between infusions, and the upper limits of cumulative exposure, and the regeneration window of the glycocalyx in real-world patient populations with diabetes, chronic kidney disease, systemic inflammation, oxidative stress, or advanced endothelial dysfunction [19]. It is likewise unknown whether repeated cycles of transient glycocalyx modulation would produce net benefit or net harm across different vascular beds.

These unknowns argue strongly for conservative, staged dose exploration and for integrating mechanistic biomarkers and safety endpoints into early clinical trials.

## 11. Translational Research Agenda

Given the early, hypothesis-generating nature of this concept, further work should proceed in a staged translational sequence. First, mechanistic preclinical studies should determine how transient hyaluronidase exposure affects glycocalyx architecture, endothelial permeability, leukocyte adhesion, thrombogenic signaling, HA-fragment generation, and lesion-level cholesterol transport. Second, translational biomarker programs should assess circulating HA species, endothelial shedding markers, inflammatory signatures, and functional readouts relevant to cholesterol efflux. Third, imaging approaches capable of probing glycocalyx integrity or vascular interface function should be incorporated where feasible. Only after these prerequisites should cautious early-phase human studies be considered.

Such early-phase studies should use conservative dose escalation, carefully defined inclusion and exclusion criteria, product-specific documentation, structured safety adjudication, and explicit stopping rules. Patients with unstable clinical presentations, uncontrolled thrombo-inflammatory disease, marked endothelial dysfunction, or substantial renal vulnerability may require exclusion or dedicated stratification in first-in-human translational settings.

## 12. Conclusions

The endothelial glycocalyx is a dynamic regulator of vascular barrier function, inflammation, and transport. Reverse cholesterol transport remains a central endogenous mechanism for removing cholesterol from arterial lesions. In this review, we propose a mechanistic hypothesis that transient systemic hyaluronidase exposure could remodel HA-containing glycocalyx and extracellular matrix structures and thereby transiently alter transport conditions relevant to cholesterol efflux.

However, the proposed link between transient glycocalyx remodeling and improved RCT remains theoretical. Direct evidence that the endothelial glycocalyx is a dominant in vivo barrier limiting HDL/ApoA-I access to plaque macrophages is currently limited, whereas the adverse consequences of glycocalyx degradation—including increased permeability, leukocyte adhesion, inflammatory signaling, and thrombo-inflammatory imbalance—are better established. The available preclinical literature is sparse and heterogeneous, and the current clinical literature consists mainly of anecdotal reports, historical uncontrolled series, and trials performed in other vascular contexts. In consequence, no convincing clinical evidence currently supports hyaluronidase therapy for chronic atherosclerosis.

Remarkably, the concept of glycocalyx renewal through hyaluronidase infusion has historical roots dating back to the 1960s, gained additional support from clinical observations in the 1980s, and may now be revitalized by results from contemporary case reports. The endothelial glycocalyx is a dynamic regulator of vascular barrier function, inflammation, and transport. RCT remains the central endogenous mechanism for eliminating cholesterol from arterial lesions.

We propose a mechanistic hypothesis that transient systemic hyaluronidase exposure could remodel HA-containing glycocalyx/extracellular matrix structures and potentially create conditions more permissive for regulated transport processes relevant to cholesterol efflux. This concept should be viewed as hypothesis-generating. It requires rigorous mechanistic validation, careful preclinical safety assessment, and only then cautiously designed clinical investigation with structured monitoring, conservative dose escalation, and explicit adjudication of adverse biological effects.

## Figures and Tables

**Table 1:** Time course of glycocalyx degradation and regeneration in relation to intravenous hyaluronidase exposure.

Process	Evidence	Time Scale	Notes
Glycocalyx thinning after hyaluronidase exposure	Rapid thinning/collapse in animal models [22,24]	Minutes	Associated with increased permeability/leukocyte adhesion
Circulating hyaluronidase activity (human PK)	Bovine testicular hyaluronidase; rHuPH20 [35,36]	t½ ≈ 2–10 min	Activity neutralized rapidly; systemic effect likely limited in duration
Early regeneration signals	recovery after enzymatic/ischemic injury [37]	hours	Reappearance of glycocalyx constituents reported experimentally
Near-complete restoration (model-dependent)	Animal/endothelial models [38,39]	~1–3 days	Strongly dependent on vascular bed and disease context
Delayed recovery states	Inflammation, hyperglycemia, sepsis [22]	Variable, potentially prolonged	Disease may impair rebuilding and function

## Data Availability

Not applicable.
